# Development of a Cell Suspension Culture System for Promoting Alkaloid and Vinca Alkaloid Biosynthesis Using Endophytic Fungi Isolated from Local *Catharanthus roseus*

**DOI:** 10.3390/plants10040672

**Published:** 2021-03-31

**Authors:** Tran My Linh, Nguyen Chi Mai, Pham Thi Hoe, Ninh Thi Ngoc, Phan Thi Hong Thao, Ninh Khac Ban, Nguyen Tuong Van

**Affiliations:** 1 Institute of Marine Biochemistry, Vietnam Academy of Science and Technology (VAST), 18 Hoang Quoc Viet, Cau Giay, Hanoi 100000, Vietnam; ncmai81@imbc.vast.vn (N.C.M.); phamthihoe93@imbc.vast.vn (P.T.H.); ninhngoc89@imbc.vast.vn (N.T.N.); ninhkhacban@vast.vn (N.K.B.); 2 Institute of Biotechnology, VAST, 18 Hoang Quoc Viet, Cau Giay, Hanoi 100000, Vietnam; pthongthaoibt@gmail.com (P.T.H.T.); vanngtg@gmail.com (N.T.V.)

**Keywords:** *Catharanthus roseus*, endophytic fungal elicitors, suspension cell culture, vinblastine

## Abstract

Cell and tissue cultures of *Catharanthus roseus* have been studied extensively as an alternative strategy to improve the production of valuable secondary metabolites. The purpose of this study was to produce *C. roseus* callus and suspension cell biomass of good quality and quantity to improve the total alkaloids and bis-indole alkaloids. The young stem derived-callus of *C. roseus* variety Quang Ninh (QN) was grown on MS medium supplemented with 1.5 mg/L 2,4-dichlorophenoxyacetic acid (2,4-D) plus 1.5 mg/L kinetin, and the growth rate increased by 67-fold after 20 days. The optimal conditions for maintaining the cell suspension culture were 150 mg/50 mL cell inoculum, a medium pH of 5.5 and a culture temperature of 25 °C. The low alkaloid content in the culture was compensated for by using endophytic fungi isolated from local *C. roseus*. Cell extracts of endophytic fungi—identified as *Fusarium solani* RN1 and *Chaetomium funicola* RN3—were found to significantly promote alkaloid accumulation. This elicitation also stimulated the accumulation of a tested bis-indole alkaloid, vinblastine. The findings are important for investigating the effects of fungal elicitors on the biosynthesis of vinblastine and vincristine, as well as other terpenoid indole alkaloids (TIAs), in *C. roseus* QN cell suspension cultures.

## 1. Introduction

*Catharanthus roseus* is well known as a medicinal plant of the Apocynaceae family that is rich in indole alkaloids. Among the 130 terpenoid indole alkaloids (TIAs) produced by the plant, bis-indole alkaloids—vincristine and vinblastine—were the first natural agents to be clinically used in the chemotherapeutic treatment of several cancers [1,2,3]. However, their concentrations in *C. roseus* plants are extremely low (0.0003–0.0004% of the dry weight of leaves), making it difficult to meet market demand [4]. Therefore, many studies have been conducted to determine how to efficiently produce these alkaloids of high medicinal value.

Plants and cultured cells are known to metabolize valuable compounds in similar ways [5]. There are examples of the successful improvement of bioactive secondary metabolites in different medical plants. The selection and development of a cell line that synthesizes and accumulates secondary metabolites depend on the appropriate explants as well as the culture conditions (nutrients, growth regulators, pH, temperature, etc.). The total triterpene production was found to be optimal when *Eriobotrya japonica* cells were cultured in MS medium instead of B5 or N6 medium [6]. A culture medium supplemented with mannitol and sucrose was found to stimulate the synthesis of total alkaloids as well as catharanthine, serpentine and ajmalicine in *C. roseus* cells [7,8]. When suspension cells were fed with loganin and tryptamine, a 17.73-fold increase in strictosidine and a 6.4-fold increase in ajmalicine were obtained [9]. A culture with a high cell density of 100 g FW/L (cell fresh weight per liter) resulted in a 2-fold increase in ajmalicine [10]. The highest biomass (0.65 g/25 mL) of *Ficus deltoidea* var. *kunstleri* was reached when using an initial inoculum size of 2.0 g/25 mL, whereas the highest flavonoid content (3.3 mg Rutin equivalents/g dried weight) was found with 0.5 g/25 mL [11]. In a suspension culture of *Eriobotrya japonica* cells, a high level of total triterpene production was obtained when the medium was supplemented with 2.5 mg/L benzyladenine (BA) and 1.0 mg/L naphthalene acetic acid (NAA) [6]. Cytokinin and ethylene have been reported to upregulate alkaloid accumulation in *C. roseus* cells through independent pathways [12]. A pH of 5.8 was found to be feasible for the production of withanolide A in cell suspension cultures of *Withania somnifera* [13]. The growth temperature of *C. roseus* plant cells for the production of ajmalicine was found to be optimal at 27.5 °C [14].

Apart from culture conditions, elicitation is also considered to be an important factor in promoting secondary metabolite biosynthesis in cell cultures. The use of fungi as elicitors for improving alkaloid production in many plants has been documented since the early 1990s [15,16]. Extracts of *Pythium*, *Botrytis*, *Phytophthora*, *Pseudomonas* and *Aspergillus* have been used as elicitors in cell suspension cultures of various medical plants, including *C. roseus* [17]. The production of catharanthine has been enhanced by using *Aspergillus niger* mycelium as an elicitor [18]. Elicitation with *Trichoderma viride* and *Phytophthora boehmeriae* resulted in a 7.9-fold increase in ajmalicine and a 4-fold increase in catharanthine in culture [19,20]. *Aspergillus flavus* promoted the production of vindoline, catharanthine and ajmaline in cambial meristematic cells of *C. roseus* by up to 1.45-, 3.29- and 2.14-fold, respectively, compared to the control [21]. 

Endophytic fungi are microfungi that are mutualistically associated with plants for at least some of their life cycle and cause no visible damage to their hosts [22]. They can be found in almost all plant species and are recognized as being important in the development of the host plant through microbe–host interactions [23]. They are also applied as elicitation factors due to their possible roles in the synthesis of active ingredients or the transformation of secondary metabolites [24]. Tang et al. [25] found that the activities of key enzymes in the alkaloid synthesis pathway, such as phenylalanine ammonia-lyase and tryptophan decarboxylase, were increased and that alkaloid production was increased by up to 48% compared to the control when using endophytic fungi as elicitors in cell suspension cultures. However, the synthesis of vinblastine and vincristine, which are important TIAs, in cultured cells has not been reported yet.

However, not all fungal elicitors work in cell culture systems because the defense systems of plant cells involve specific receptors that only recognize specific elicitors [26]. Therefore, it is essential to assess different elicitors to maximize the production of a desired compound in a particular system. 

In this study, the effects of plant growth regulators on callus initiation, multiplication and alkaloid content were evaluated. A suitable cell suspension culture for the production of alkaloids and tested bis-indole alkaloid—vinblastine—was established based on the optimization of the initial size of the inoculum, the pH of the medium, culture temperature and elicitation by endophytic fungi isolated from a local *C. roseus* variety. 

## 2. Results

### 2.1. Callus Initiation and Proliferation from Local C. roseus

The explants used for callus formation in this study were stem fragments of 10-day-old germinated plants. Callus induction was evaluated using different concentrations of the auxins 2,4-dichlorophenoxyacetic acid (2,4-D), indole-3-acetic acid (IAA) and naphthalene acetic acid (NAA). The obtained results clearly indicate that 2,4-D strongly promoted the formation of high-quality (soft and fragile) calli at concentrations higher than 1.0 mg/L. The number of callus-forming explants increased when the 2,4-D concentration was increased from 0.5 to 2.0 mg/L (Table 1). At a 2,4-D concentration higher than 2.0 mg/L, the color of the calli changed to brown after 3 weeks. The addition of NAA at 0.5–2.5 mg/L promoted the production of hairy roots from explants, and only 2–3% of explants formed hard calli after 21 days. With IAA, all of the explants expanded at the cut ends of the stem fragments, but they gradually died without forming calli (data not shown). Without growth regulators, 50% of the explants died after 28 days of culture. 

**Figure 1:**
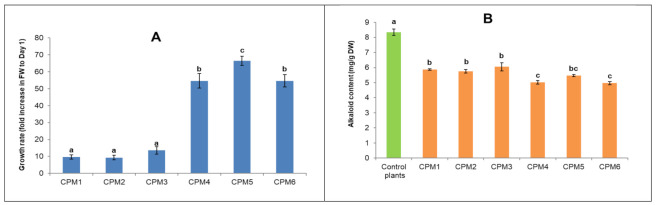
Growth rates (**A**) and total alkaloid contents (**B**) of calli multiplied in different CPMs. The bars and error bars show the means and the standard errors, respectively. Different letters (a, b, c) show significant differences at *p* < 0.05.

Callus multiplication was carried out in solid callus proliferation medium (CPM) supplemented with different concentrations of 2,4-D or a combination of 2,4-D and kinetin. Growth rate analysis of calli in CPMs with different plant growth regulator formulas revealed that calli could not multiply effectively in media with 2,4-D only (CPM1 to CPM3) (Figure 1A). In these cases, the fresh weight (FW) of calli only increased by around 10-fold after 20 days of culture, while there was more than a 60-fold increase in FW for calli cultured in the media containing 2,4-D plus kinetin, namely, CPM4, CPM5 and CPM6. The most effective combination was 1.5 mg/L kinetin and 1.5 mg/L 2,4-D (CPM5), in which the FW was increased by 67-fold after 20 days (from 0.057 to 3.794 g). The proliferation of the calli slowly decreased from the 21st day onwards. 

The results of total alkaloid analysis, as shown in Figure 1B, indicate that the alkaloid accumulation in the calli was significantly lower than that in the natural plants. The alkaloid content of *C. roseus* Quang Ninh (QN) collected in nature was 8.35 milligrams per gram of dried weight (DW), while the highest alkaloid content obtained from the calli on CPM3 was 6.03 milligrams per gram of DW (Figure 1B). In addition, the alkaloid levels in CPMs were found to be opposite to the callus growth rates. The alkaloid contents of calli that multiplied in the low growth rate media (CPM1, CPM2 and CMP3) were higher than those in the higher growth rate ones (CPM4, CPM5 and CPM6) (Figure 1). 

### 2.2. Cell Suspension Establishment

**Figure 2:**
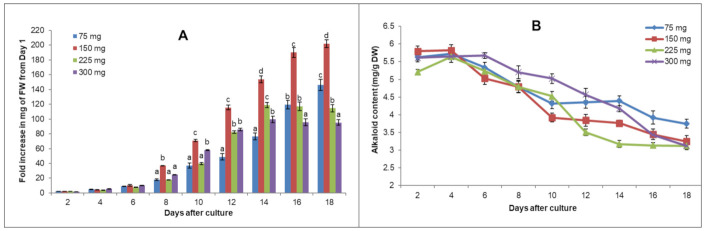

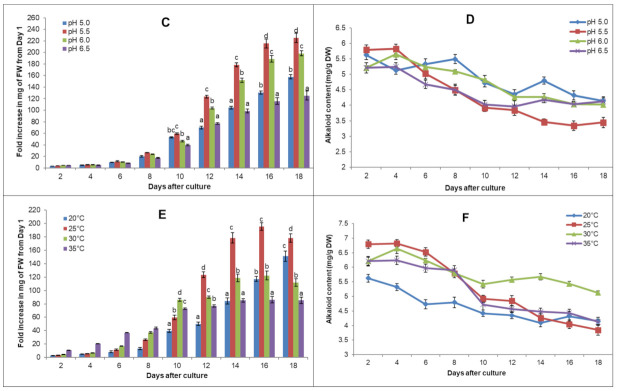
Effects of initial inoculum, pH of the medium and culture temperature on growth kinetics and alkaloid accumulations of C. roseus QN cell suspension cultures. (**A**,**B**) Growth rate and total alkaloid content using 75, 150, 225 and 300 mg cell inoculum/50 mL; (**C**,**D**) Growth rate and Table 5.0, 5.5, 6.0 and 6.5; (**E**,**F**) Growth rate and total alkaloid content of cells cultured at temperatures of 20, 25, 30 and 35 °C. The error bars show the standard errors. Different letters (a, b, c, d) show significant differences at *p* < 0.05 among CPMs on the same harvested date.

The suspension cultures were initiated by adding different cell inoculum sizes from the suspension stock. The fold increase from Day 1 was calculated based on the fresh weight, determined every 2 days. The results shown in Figure 2A indicate that in all cases, the log phase fell in the first 8 days. The log phase periods varied from 4 days (10th to 14th day) for 220 and 300 mg and 6 days (10th to 16th day) for 150 mg to more than 8 days for 75 mg. The fastest and highest biomass increase was obtained from 150 mg of initial cells; this culture exhibited a 190-fold increase in biomass after 16 days, while the increases were 110- and 95-fold for 220 and 300 mg of initial cells, respectively. By the 16th day, although still in the log phase, the culture starting with 75 mg of cells only showed a 119-fold increase. Consequently, 150 mg of cells from the suspension stock was chosen for the subsequent experiments.

Important factors affecting the cell suspension cultures, including the pH of the medium and temperature, were tested. The culture at pH of 5.5 was better in promoting cell growth than the original pH of 6.0 (Figure 2C). Biomass increases of up to 215-fold were obtained after 16 days of culture, while they were 190-fold for pH 6.0 and around 120-fold for pH values of 5.0 and 6.5. A temperature of 25 °C was found to be ideal; higher temperatures (30 and 35 °C) appeared to inhibit cell division and significantly reduce the obtained biomass (Figure 2E,F). A temperature of 20 °C might prolong cell division cycles, lengthening the log phase period. Therefore, the optimal cell suspension system was determined to involve the high-alkaloid local *C. roseus* variety QN, starting with 150 mg of cells and culturing in 100 mL of CPM medium at pH 5.5 and 25 °C.

The alkaloid contents of each sample were also analyzed. The obtained results shown in Figure 2B,D,F indicate that cells in suspension culture metabolized alkaloids in lower amounts than those in plants and calli. As observed in the calli as well in suspended cells, the alkaloid accumulation was related to the culture growth rate. For example, the alkaloid content in cells grown in the optimal temperate of 25 °C was reduced from 6.793 to 3.844 mg/DW (43.4% lower) after 18 days, while there was 26.2%, 17.4% and 28.7% decrease in the lower growth cultured condition of 20 °C, 30 °C and 35 °C, respectively. Therefore, the best conditions for maximizing biomass were not optimal for alkaloid production. 

### 2.3. Endophytic Fungus Isolation and Elicitation

**Figure 3:**
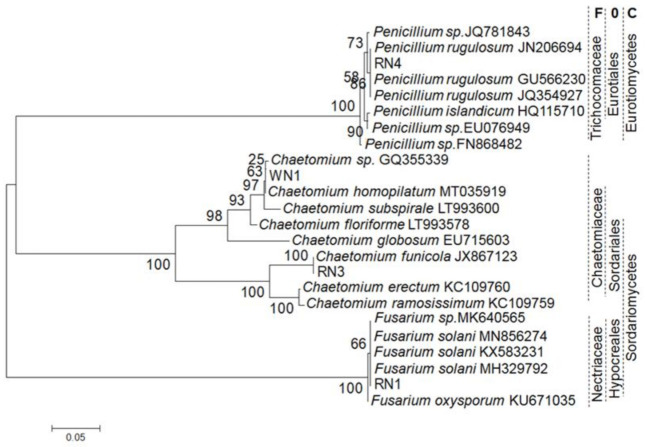
Phylogenetic neighbor-joining tree reconstructed based on DNA polymorphisms of ITS fragments of four endophytic isolates and referenced taxa. F, family; O, order; C, class. Numbers after taxa are GenBank accession numbers. Numbers next to nodes of clades are bootstrap values (%). RN1, RN3, RN4 and WN1: names of isolates.

Four isolates were obtained from root samples of *C. roseus*; three (named RN1, RN3 and RN4) were from a pink-flowered plant, and the fourth (named WN1) was from a white-flowered plant. Phylogenetic analyses based on internal transcribed spacer (ITS)-amplified fragments from the isolates and referenced taxa showed that they were fungi (Figure 3) belonging two classes, three orders, three families and three genera in three clades of the tree with a bootstrap value of 100%. In the neighbor-joining tree, RN1 belongs to the *Fusarium* genus, Nectriaceae family, Hypocreales order and Sordariomycetes class, while RN3 and WN1 both belong to the *Chaetomium* genus, Chaetomiaceae family, Sordariales order and Sordariomycetes class. RN4 is in a clade within the taxa of the *Penicillium* genus. The bootstrap values were 66, 25–100 and 58–90, respectively, within each clade of the *Fusarium*, *Chaetomium* and *Penicillium* genera. 

The alignment between the DNA sequences of the ITS fragments of the four isolates and four referenced taxa in each clade (Figure 3) revealed that the homology and genetic distance ranged from 64.5 to 100% and 0.000 to 0.361, respectively (Table 2). RN1 was classified as *Fusarium solani*, while RN3 and WN1 were *Chaetomium funicola* and *Chaetomium homopilatum;* RN4 was identified as *Penicillium rugulosum*. Four pair-alignments of the ITS fragments of the isolates and referenced taxa all showed maximum homology levels (100%) and minimum genetic distances (0.000).

**Figure 4:**
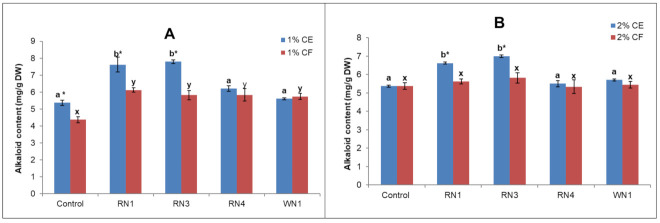
Alkaloid contents under elicitation with endophytic fungus cell extracts (CE) and filtrates (CF). (**A**) Elicitation with 1% CE or CF; (**B**) elicitation with 2% CE or CF. RN1: *Fusarium solani* RN1 isolate: RN3: *Chaetomium funicola* RN3 isolate; RNA4: *Penicillium rugulosum* RN4 isolate and WN1: *Chaetomium homopilatum* WN1. Control: cell culture without treatment of endophytic fungi. The error bars show the standard errors. Means with the same letter did not show significant differences in total alkaloid contents; asterisks indicate significant differences at * *p* < 0.05 between CE and CF of one treatment source.

The alkaloid contents of suspension cells treated with endophytic fungi were found to depend on the elicitor preparation (cell extract (CE) or culture filtrate (CF)) and fungal species. Alkaloid accumulation in the cultured suspension cells treated with *Fusarium solani* RN1 and *Chaetomium funicola* RN3 significantly increased compared to the control (Figure 4). The CE elicitors appeared to have stronger effects than CF at both tested concentrations. The highest alkaloid contents of 7.6 and 7.7 mg/g DW were obtained from the cultures elicited with 1% CE of RN1 and RN3, respectively, followed by those treated with 2% CE (6.6–6.9 mg/g DW). 

**Figure 5:**
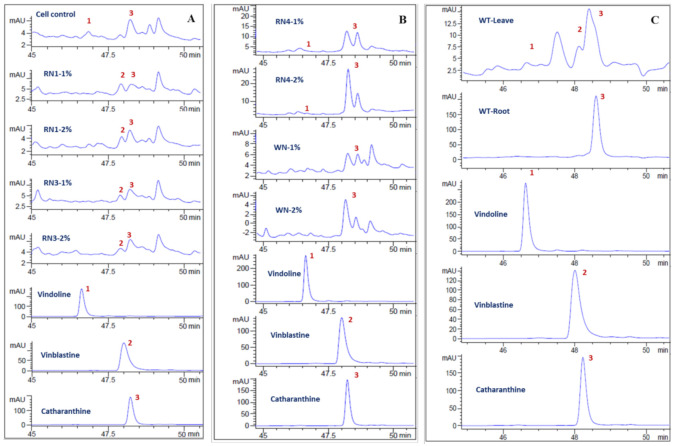
Chromatograms of vindoline (1), vinblastine (2) and catharanthine (3) identified in the leaves, roots and suspension cells elicited with endophytic fungal extracts. (**A**) RN1 and RN3; (**B**) RN4 and WN1; (**C**) leaves and roots of the wild type (WT).

The production of vindoline, catharanthine and vinblastine in the extracts of suspension cells elicited with the CE of each fungal isolate (RN1, RN3, RN4 and WN1) was analyzed. Using chromatograms (Figure 5), the compounds in the extracts were identified based on their retention times compared to standards, and different compounds were found among the elicitor sources. Although vinblastine and its two precursors—vindoline and catharanthine—were produced in *C. roseus* leaves (Figure 5C), vinblastine was not observed in the cell suspension control (Figure 5A, cell control). Most interesting is that treating *C. roseus* suspension cultures with 1 or 2% concentrations of the CE of RN1 and RN3 resulted in vinblastine accumulation (Figure 5A). The CE of RN4 did not appear to affect vinblastine biosynthesis (Figure 5B). The WN1 cell extract actually had a negative impact on TIA biosynthesis, as evidenced by the absence of vindoline. 

## 3. Discussion

In this study, calli and cell suspension cultures of local *C. roseus* QN were established. A suspension culture cycle of 16 days was optimal for biomass production. Although the fresh weight of cultured cells was dramatically increased (up to 200 times in one cycle), the alkaloid contents were significantly reduced compared to those of the plants and even the calli. The culture conditions that favored rapid cell division rates seem to have affected secondary metabolite synthesis. Van Der Heijden et al. [27] observed a significant decrease in cell growth but a remarkable increase in TIA accumulation upon reducing or removing nitrate and/or phosphate ions from the medium. This phenomenon was also reported by Smetanska [28], who found that the synthesis of secondary metabolites in cell cultures was affected differently from that in plants and depended on environmental conditions. Another reason may be the use of 2,4-D in the calli and cell cultures of *C. roseus* QN. It was the best auxin for stimulating callus formation and division, but it is also considered to be an inhibitor of alkaloid and indole biosynthesis [7,29].

For elicitation, four species of endophytic fungi isolated from local *C. roseus* were identified based on ITS regions, which are a common barcode marker for resolving phylogenetic relationships among different genera. *Trichocladium opacum*, which used to belong to the *Trichocladium* genus of the Chaetomiaceae family, was transferred to another genus—*Pleotrichocladium* of the Melanommataceae family—when an ITS marker was employed [30]. It is believed that the endophytic fungi isolated from the local *C. roseus* in this study are *Fusarium solani*, *Chaetomium funicola*, *Penicillium rugulosum* and *Chaetomium homopilatum*. In the past, eight different fungal genera from the leaves of *C. roseus*—including *Alternaria*, *Aspergillus*, *Curvularia*, *Penicillium*, *Trichoderma*, *Helminthosporium*, *Fusarium* and *Phoma*—were reported [31]. Other genera—*Chaetomium*, *Colletotrichum*, *Dothideomycetes*, *Eutypella*, *Eutypa*, *Flavodon*, *Talaromyces*, *Choanephora*, *Lasiodiplodia*, *Cophinforma*, *Macrophomina* and *Nigrospora*—were found in different parts of the plant [32,33,34,35]. However, endophytic fungi exploited as elicitors in *C. roseus* tissue culture are few. 

The low alkaloid accumulation in our cell suspension culture system was corrected by elicitation using *C. roseus* endophytic fungi. The elicitation stimulated the biosynthesis of total alkaloids and vinblastine. Vinblastine was selected for the analysis of bis-indole in this study because its content is usually higher than vincristine in the plant and vincristine is transformed from vinblastine [36]. In the past, cell suspension cultures of *C. roseus* have been employed for the production of TIAs and many other pharmaceutically important products [37]; however, one of the major disadvantages of this approach is the inability of these cultures to produce vindoline and, hence, vinblastine and vincristine [38,39]. According to Hussain et al. [40], the production of vincristine or vinblastine from *C. roseus* in cell cultures is distinct and may require a certain degree of differentiation before a product can be synthesized. Therefore, elicitors are critical for improving TIA production in cell culture suspension systems. 

The CEs of *Fusarium solani* RN1 and *Chaetomium funicola* RN3 were shown to be the most effective elicitors. The effects of RN1, RN3 and RN4 differed from that of WN1, which may be because the former isolates were from *C. roseus* var. *roseus* (known as high alkaloids in our preliminary experiments) and the latter was from *C. roseus* var. *ocellatus* (low alkaloids). Cell extracts of microbes are known to be rich in polysaccharide and peptide moieties [41], which have been recognized as signaling elements in the enrichment of secondary metabolites [42]. According to Abdul Malik [43], plant cells have receptors on their plasma membranes that are responsible for the recognition of fungal elicitors and transfer information that activates the plant’s cell defense system. 

## 4. Materials and Methods

### 4.1. Explant Preparation, Callus Initiation and Induction

Wild plants were collected from the beaches of Quang Ninh province, Vietnam (QN) in the summer of 2018 for alkaloid content analysis and mature seeds. The seeds were dried at room temperature (30 °C) and surface sterilized with 2% sodium hypochlorite for 5 min, washed with sterile distilled water and germinated on MS medium [44] containing 30 mg/L sucrose and 8% agar at pH 5.7. Germination was carried out at 25 °C in darkness. 

For callus formation, 5 mm stem fragments of in vitro germinated plants were placed on solid callus induction medium (CIM) containing 30 g/L sucrose, MS salts and vitamins supplemented with 0.5, 1.0, 1.5, 2.0 and 2.5 mg/L of IAA or NAA or 2,4-D. After four weeks, the induced calli were detached from the explants, and their efficiencies were compared based on fresh weight. In order to determine the best formulation for callus proliferation, the calli initiated from the explants were divided into 2 × 2 mm^2^ pieces and placed on callus proliferation solid medium (CPM). The CPMs contained nutrition as CIM supplemented with 1.0, 1.5 and 2.0 mg/L 2,4-D only (designated as CPM1, CPM2 and CPM3, respectively) or 1.5 mg/kinetin combined with 1.0, 1.5 and 2.0 mg/L 2,4-D (designated as CPM4, CPM5 and CPM6, respectively). After 20 days, the growth rate was determined by the fold increase in fresh weight and total alkaloids of calli in each culture and compared. A total of 10 Petri dishes (10 calli/dish) were cultured for each treatment, and the experiments were repeated thrice. 

### 4.2. Establishing Cell Suspension Cultures

To establish the cell suspension stock, a callus growing rapidly on the CPM was selected and placed in a 250 mL flask containing 50 mL of liquid CPM with pH 6 and incubated at room temperature (25 °C) on a rotary shaker at 120 rpm. The cell suspension cultures were subcultured every two weeks using a sieve to homogenize them and facilitate more cell suspension experiments. The culture system was optimized by monitoring the kinetics of cell growth based on differences in cell inoculum sizes (75, 150, 225 and 300 mg/50 mL), pH of the medium (5.0, 5.5, 6.0 and 6.5) and culture temperatures (20, 25, 30 and 35 °C). The cell inoculum experiment was first cultured in liquid CPM at pH 6.0 and temperature of 25 °C. The optimal inoculum and then pH were used for testing culture temperature. The cell growth in the suspensions was calculated based on the fresh weight (mg) collected every 2 days after filtering using Whatman No. 1 filter paper. For alkaloid analysis, cells were dried in an oven at 50 °C until a constant weight was reached. 

### 4.3. Isolation of Endophytic Fungi and Taxonomy Identification 

Roots of *C. roseus* var. *roseus* (pink flowers) and *C. roseus* var. *ocellatus* (white flowers) were collected from wild-type plants growing along the coast of Nha Trang, Khanh Hoa, Vietnam. The root samples were washed thoroughly with tap water and then cut into 1-cm-long pieces. They were surface sterilized by soaking them in 70% ethanol for 1 min and then in 5% sodium hypochlorite (NaOCl) for 1 min, after which they were rinsed 5 times with sterile distilled water. The sterilized roots were homogenized in a mortar and pestle, and then the liquid was collected and spread on Czapek medium. After incubation for 7–14 days at 25 °C, typical fungal colonies were identified. 

Molecular methods were used to confirm the identity of the endophytic isolates. The total DNA of each isolate was obtained from the biomass on non-sporulating agar medium using a DNeasy Plant Mini Kit (Qiagen, Hilden, Germany). Internal transcribed spacer (ITS) ribosome DNA regions were amplified using the fungus-specific primers ITS1 (5′-TCC GTA GGTGAA CCT GCG G-3′) and ITS4 (5′-TCC TCC GCT TAT TGA TAT GC-3′) [45]. The mixture was then subjected to thermal cycling (Eppendorf, Germany) for PCR, beginning with an initial activation step at 95 °C for 15 min to activate the DreamTaq DNA polymerase (Thermo Fisher Scientific, Lithuania). The following cycle was repeated 35 times: denaturing for 1 min at 95 °C, annealing for 1 min at 56 °C, extension for 1 min at 72 °C and a final extension for 10 min at 72 °C. The amplified ITS regions (586–600 bp) were sequenced and analyzed for phylogeny using Neighbor Joining (NJ) method on Mega 3.1 with Kimura 2-parameter model of 1000 replicates. Homology-level analysis for species identification was conducted using Multiple DNA Sequence Alignment in DNAMAN4.15 based on the homology level (%) and distance matrix (<1.000).

### 4.4. Elicitor Preparation and Application

The fungal elicitors (cell extract and culture filtrate) were prepared according to the procedure described by Baldi et al. [46] with some modifications. Fungal mycelia were collected after 7 days of culturing on 100 mL of Hansen medium at 30 °C in a 250 mL flask agitated at 200 rpm. The mycelia were separated from the broth by filtration and washed several times with double-distilled water. They were then dried at 65 °C until constant weights were obtained and then powdered in liquid nitrogen. About 10 g of cell powder was suspended in 100 mL of double-distilled water and autoclaved at 120 °C for 15 min. The supernatant obtained after centrifuging the suspension at 5000 rpm for 20 min was used as the cell extract (CE). The culture broth was passed through a 0.22 µm filter and used as the filtered culture broth (CF). 

For the elicitation treatments, the cultures were elicited with 1.0 and 2.0% (*v/v*) concentrations of the CE and CF of each fungal elicitor on the 14th day. The control received an equal volume of Czapek medium and water instead of the CF and CE, respectively. The cultures were harvested on the 17th day and analyzed for total alkaloids or vindoline, catharanthine and vinblastine production. 

### 4.5. Extraction and Determination of Total Alkaloids 

The total alkaloids were determined using the method described by Gomaa et al. [47] with minor modifications. The dried *C. roseus* samples were powdered, extracted three times with EtOH (with a 1:25 ratio of sample mass/ethanol volume) and then sonicated for 20 min at 45 °C. The EtOH extract was diluted with water, and the pH was adjusted to 1 with 38% HCl; extraction was then performed with hexane (1:1, *v/v*) for 2 h at room temperature (RT). The upper phase was removed, and the lower aqueous phase was basified to pH 8.0 with 1 M NH_4_OH and then extracted with dichloromethane (1:1, *v/v*) for 2 h at RT. The dichloromethane layer contained basic alkaloids while the aqueous phase contained NH_4_ ions. The organic phase was collected and vacuum dried at 55 °C to afford the crude alkaloids. To quantify the total alkaloids, the dried residue of each sample was weighed using an analytical balance; the resulting mass of alkaloids per gram of dried *C. roseus* sample was noted. Each experiment was carried out in triplicate. 

### 4.6. HPLC Analysis

The culture cells were harvested, washed twice with distilled water and dried at 50 °C in a dry-heat oven until a constant weight was achieved. A quantity of 5 g of the dried sample was extracted with MeOH (150 mL) and then sonicated for 30 min at 30 °C. The MeOH crude extract was diluted with 25 mL of water, and the pH was adjusted to 1.0 with ~25 µL of 38% HCl, followed by extraction with 25 mL of hexane. The aqueous phase was collected, basified to pH 8.0 with 1 M NH_4_OH and then extracted with ethyl acetate (25 mL) to afford the crude alkaloid extract for HPLC analysis. For the wild-type plants, 50 g of dried samples (roots or leaves) were powered and extracted using the same procedure.

Chromatography was performed by using an HPLC Agilent Technologies 1200 Series system with gradient elution on an Agilent Eclipse XDB-C18 column (4.6 × 250 mm; 5 µm). The mobile phase consisted of a 25 mM aqueous ammonium acetate solution (Solvent A) and acetonitrile (Solvent B). The following program was used for alkaloid separation: a linear gradient from 10 to 90% Solvent B for 45 min, 90% Solvent B for 15 min and then holding at 10% Solvent B for 15 min before a new injection. Sample aliquots of 10 µL were injected and eluted from the column with a flow rate of 500 µL per min. The column was maintained at room temperature (25.3–26.7 °C). A DAD detector was employed for the detection of peaks, set at wavelengths of 210–365 nm. The concentrations were 2 mg/mL for the extracts and 0.2 mg/L for the purified agents. The flow rate was 0.5 mL/min, and the injection volume was 10 μL. Catharanthine (#116M4718V, Sigma-Aldrich, China), vinblastine sulfate (#078M4002V, Sigma-Aldrich, Israel) and purified vindoline, generously provided by the Department of Marine Medicinal Materials, Institute of Marine Biochemistry-VAST, were used as standards.

### 4.7. Statistical Analysis

The growth rate of callus, suspension cells and alkaloid contents were presented as the mean of three replicates ± standard error using Microsoft Excel 2010. Duncan’s multiple range test was employed to analyse differences between the means using IBM SPSS Statistics for Windows, Version 20.0 (Armonk, NY: IBM Corp.). *p* value < 0.05 was considered statistically significant.

## 5. Conclusions

In this study, a quality callus for suspension culture of cells was established from stem fragments of *C. roseus* variety QN and multiplied on MS medium supplemented with 1.5 mg/L kinetin and 1.5 mg/L 2,4-D. Cell suspension cultures were maintained by subculturing using 150 mg initial cell inoculum/50 mL, a medium pH of 5.5 and culture temperature of 25 °C. The low alkaloid accumulation in this system was compensated for by using cell extracts of the endophytic fungi *F. solani* RN1 and *C. funicola* RN3. The most important finding is that the fungal treatments elicited the accumulation of one tested bis-indole alkaloid, vinblastine. This finding is of importance for further investigating the contribution of our fungal elicitors to the biosynthesis of two bis-indole alkaloids—vinblastine and vincristine—as well as other TIAs.

## Figures and Tables

**Table 1:** Effects of NAA and 2,4-D on callus initiation from stem fragments of *C. roseus* QN.

Plant Growth Regulators (mg/L)		Percentage of Explants Forming Callus (%)			
		Day 7	Day 14	Day 21	Day 28
**2.4-D**	0.5	16.7 ± 1.87 ^a^	46.3 ± 2.12 ^a^	83.0 ± 1.88 ^a,b^	83 ± 2.34 ^b^
	1	53.0 ± 2.00 ^b^	90.3 ± 3.02 ^b,c^	93.0 ± 2.19 ^b,c^	100.0 ± 0.0 ^c^
	1.5	58.7 ± 1.26 ^b^	97.7 ± 1.76 ^c^	100.0 ± 0.0 ^c^	100.0 ± 0.0 ^c^
	2	65.3 ± 2.73 ^b,c^	97.3 ± 4.44 ^c^	100.0 ± 0.0 ^c^	100.0 ± 0.0 ^c^
	2.5	70.0 ± 1.76 ^c^	86.7 ± 1.30 ^b^	77.7 ± 3.35 ^a^	68.9 ± 2.17 ^a^
**NAA**	0.5	0	0	0	0
	1	0	0	0	0
	1.5	0	0	3.3 ± 0.53	3.3 ± 0.12
	2	0	0	2.2 ± 0.21	2.2 ± 0.39
	2.5	0	0	0	0

Data are means ± SE (*n* = 3 biological replicates). Different letters (a, b, c) in one column show significant differences at *p* ˂ 0.05.

**Table 2:** Alignment for homology between ITS fragments of four isolates and referenced fungal species.

#	Taxa	1	2	3	4	5	6	7	8
		Genetic Distance (0–1)							
1	*C. funicola* JX867123		0.104	0.239	0.361	0.239	0.000	0.361	0.104
2	*C. homopilatum* MT035919	89.6		0.232	0.355	0.232	0.104	0.355	0.000
3	*F. solani* MH329792	76.1	76.8		0.313	0.000	0.239	0.313	0.232
4	*P. rugulosum* JN206694	63.9	64.5	68.7		0.313	0.361	0.000	0.355
5	RN1	76.1	76.8	100.0	68.7		0.239	0.313	0.232
6	RN3	100.0	89.6	76.1	63.9	76.1		0.361	0.104
7	RN4	63.9	64.5	68.7	100.0	68.7	63.9		0.355
8	WN1	89.6	100.0	76.8	64.5	76.8	89.6	64.5	
		**Homology level (%)**							

## Data Availability

Not Applicable.

## References

[1] Noble R.L. (1990). The discovery of the vinca alkaloids—Chemotherapeutic agents against cancer. Biochem. Cell Biol..

[2] Shams K.A., Nazif N.M., Azim N., Shafeek K., Missiry M., Ismail S., Nasr M. (2009). Isolation and characterization of antineoplastic alkaloids from *Catharanthus roseus* L. Don. cultivated in Egypt. Afr. J. Tradit. Complement. Altem. Med..

[3] Van Der Heijden R., Jacobs D., Snoeijer W., Hallard D., Verpoorte R. (2004). The Catharanthus Alkaloids: Pharmacognosy and Biotechnology. Curr. Med. Chem..

[4] Barrales-Cureño H.J. (2015). Pharmacological applications and in vitro biotechnological production of anticancer alkaloids of *Catharanthus roseus*. Biotechnol. Apl..

[5] Hellwig S., Drossard J., Twyman R.M., Fischer R. (2004). Plant cell cultures for the production of recombinant proteins. Nat. Biotechnol..

[6] Ho H.Y., Liang K.Y., Lin W.C., Kitanaka S., Wu J.B. (2010). Regulation and improvement of triterpene formation in plant cultured cells of *Eriobotrya japonica* Lindl. J. Biosci. Bioeng..

[7] Zhao J., Zhu W.H., Hu Q., He X.W. (2001). Enhanced indole alkaloid production in suspension compact callus clusters of *Catharanthus roseus*: Impacts of plant growth regulators and sucrose. Plant Growth Regul..

[8] Mishra M.R., Srivastava R.K., Akhtar N. (2018). Enhanced alkaloid production from cell culture system of *Catharanthus roseus* in combined effect of nutrient salts, sucrose and plant growth regulators. J. Biotechnol. Biomed. Sci..

[9] El-Sayed M., Verpoorte R. (2002). Effect of phytohormones on growth and alkaloid accumulation by a *Catharanthus roseus* cell suspension cultures fed with alkaloid precursors tryptamine and loganin. Plant Cell Tiss. Organ. Cult..

[10] Lee C.W.T., Shuler M.L. (2000). The effect of inoculum density and conditioned medium on the production of ajmalicine and catharanthine from immobilized *Catharanthus roseus* cells. Biotechnol. Bioeng..

[11] Haida Z., Syahida A., Ariff S.M., Maziah M., Hakiman M. (2019). Factors affecting cell biomass and flavonoid production of *Ficus deltoidea* var. kunstleri in cell suspension culture system. Sci. Rep..

[12] Yahia A., Kevers C., Gaspar T., Chénieux J.C., Rideau M., Crèche J. (1998). Cytokinins and ethylene stimulate indole alkaloid accumulation in cell suspension cultures of *Catharanthus roseus* by two distinct mechanisms. Plant Sci..

[13] Nagella P., Murthy H.N. (2010). Establishment of cell suspension cultures of *Withania somnifera* for the production of withanolide A. Bioresour. Technol..

[14] Hoopen H.J.G., Vinke J.L., Moreno P., Verpoorte R., Heijnen S. (2002). Influence of temperature on growth and ajmalicine production by *Catharanthus roseus* suspension cultures. Enzyme Microb. Technol..

[15] Christen A., Gibson D., Bland T. (1991). Production of Taxol or Taxol-Like Compounds in cell Culture. US Patent.

[16] Strobel G.A., Stierle A., van Kuijk F.J.G.M. (1992). Factors influencing the in vitro production of radiolabeled taxol by Pacific yew, *Taxus brevifolia*. Plant Sci..

[17] Tonk D., Mujib A., Maqsood M., Ali M., Zafar N. (2016). *Aspergillus flavus* fungus elicitation improves vincristine and vinblastine yield by augmenting callus biomass growth in *Catharanthus roseus*. Plant Cell Tiss. Organ Cult..

[18] Zhao J., Hu Q., Guo Y.Q., Zhu W.H. (2001). Effects of stress factors, bioregulators, and synthetic precursors on indole alkaloid production in compact callus clusters cultures of *Catharanthus roseus*. Appl. Microbiol. Biotechnol..

[19] Namdeo A., Patil S., Fulzele D.P. (2002). Influence of fungal elicitors on production of ajmalicine by cell cultures of *Catharanthus roseus*. Biotechnol. Prog..

[20] Chen Q., Chen Z., Lu L., Jin H., Sun L., Yu Q., Xu H., Yang F., Fu M., Li S. (2013). Interaction between abscisic acid and nitric oxide in PB90-induced catharanthine biosynthesis of *Catharanthus roseus* cell suspension cultures. Biotechnol. Prog..

[21] Liang C., Chen C., Zhou P., Xu L., Zhu J., Liang J., Zi J., Yu R. (2018). Effect of *Aspergillus flavus* fungal elicitor on the production of terpenoid indole alkaloids in *Catharanthus roseus* cambial meristematic cells. Molecules.

[22] Abdel-Azeem A.M., Abdel-Azeem M.A., Khalil W.F., Watson R.R., Preedy V.R. (2019). Endophytic fungi as a new source of antirheumatoid metabolites. Bioactive Food as Dietary Interventions for Arthritis and Related Diseases.

[23] Kaul S., Ahmed M., Sharma T., Dhar M.K., Kharwar R.N., Upadhyay R., Dubey N., Raghuwanshi R. (2014). Unlocking the myriad benefits of endophytes: An overview. Microbial Diversity and Biotechnology in Food Security.

[24] Boller T. (1995). Chemoperception of microbial signals in plant cells. Annu. Rev. Plant Biol..

[25] Tang Z., Rao L., Peng G., Zhou M., Guorong S., Liang Y. (2011). Effects of endophytic fungus and its elicitors on cell status and alkaloid synthesis in cell suspension cultures of *Catharanthus roseus*. J. Med. Plants Res..

[26] Barrett L., Heil M. (2012). Unifying concepts and mechanisms in the specificity of plant-enemy interactions. Trends Plant Sci..

[27] Van Der Heijden R., Verpoorte R., Ten Hoopen H.J.G. (1989). Cell and tissue cultures of *Catharanthus roseus* (L.) G. Don: A literature survey. Plant Cell Tiss. Organ. Cult..

[28] Smetanska I. (2008). Production of secondary metabolites using plant cell cultures. Food Biotechnol..

[29] Gantet P., Imbault N., Thiersault M., Doireau P. (1997). Inhibition of alkaloid accumulation by 2,4-D in *Catharanthus roseus* cell suspension is overcome by methyl jasmonate. Acta Bot. Gall..

[30] Hernández-Restrepo M., Gené J., Castañeda-Ruiz R., Mena-Portales J., Crous P., Guarro J. (2017). Phylogeny of saprobic microfungi from Southern Europe. Stud. Mycol..

[31] Momsia P., Momsia T. (2013). Isolation, frequency distribution and diversity of novel fungal endophytes inhabiting leaves of *Catharanthus roseus*. Int. J. Life Sci. Biotechnol. Pharm. Res..

[32] Palem P.P.C., Kuriakose G.C., Jayabaskaran C. (2015). An endophytic fungus, *Talaromyces radicus*, isolated from *Catharanthus roseus*, produces vincristine and vinblastine, which induce apoptotic cell death. PLoS ONE.

[33] Pandey S.S., Singh S., Babu C.S.V., Shanker K., Srivastava N.K., Shukla A.K., Kalra A. (2016). Fungal endophytes of *Catharanthus roseus* enhance vindoline content by modulating structural and regulatory genes related to terpenoid indole alkaloid biosynthesis. Sci. Rep..

[34] Ayob F., Simarani K. (2016). Endophytic filamentous fungi from a *Catharanthus roseus*: Identification and its hydrolytic enzymes. Saudi Pharm. J..

[35] Dhayanithy G., Subban K., Chelliah J. (2019). Diversity and biological activities of endophytic fungi associated with *Catharanthus roseus*. BMC Microbiol..

[36] Pham H.N., Vuong Q.V., Bowyer M.C., Scarlett C.J. (2020). Phytochemicals derived from *Catharanthus roseus* and their health benefits. Technologies.

[37] Tikhomiroff C., Jolicoeur M. (2002). Screening of *Catharanthus roseus* secondary metabolites by high-performance liquid chromatography. J. Chromatogr. A.

[38] St-Pierre B., De Luca V.A. (1995). Cytochrome P-450 monooxygenase catalyzes the first step in the conversion of tabersonine to vindoline in *Catharanthus roseus*. Plant Physiol..

[39] Vazquez-Flota F., De Carolis E., Alarco A.M., De Luca V. (1997). Molecular cloning and characterization of desacetoxyvindoline-4-hydroxylase, a 2-oxoglutarate dependent-dioxygenase involved in the biosynthesis of vindoline in *Catharanthus roseus* (L.) G. Don. Plant Mol. Biol..

[40] Hussain M.S., Fareed S., Ansari S., Rahman M.A., Ahmad I.Z., Saeed M. (2012). Current approaches toward production of secondary plant metabolites. J. Pharm. Bioallied Sci..

[41] Ruiz-Herrera J., Ortiz-Castellanos L. (2019). Cell wall glucans of fungi. A review. Cell Surf..

[42] Zhao J., Davis L.C., Verpoorte R. (2005). Elicitor signal transduction leading to production of plant secondary metabolites. Biotechnol. Adv..

[43] Abdul Malik N.A., Kumar I.S., Nadarajah K. (2020). Elicitor and receptor molecules: Orchestrators of plant defense and immunity. Int. J. Mol. Sci..

[44] Murashige T., Skoog F. (1962). A revised medium for rapid growth and bioassays with tobacco cultures. Physiol. Plant..

[45] White T.J., Bruns T., Lee S., Taylor J., Innis M.A., Gelfand D.H., Sninsky J.J., White T.J. (1990). Amplification and direct sequencing of fungal ribosomal RNA genes for phylogenetics. PCR Protocols: A Guide to Methods and Applications.

[46] Baldi A., Srivastava A., Bisaria V.S., Varma A., Kharkwal A.C. (2009). Fungal elicitors for enhanced production of secondary metabolites in plant cell suspension cultures. Symbiotic Fungi Soil Biology.

[47] Gomaa N.H., Hassan M.O., Fahmy G.M., González L., Hammouda O., Atteya A.M. (2014). Allelopathic effects of *Sonchus oleraceus* L. on the germination and seedling growth of crop and weed species. Acta Bot. Bras..

